# Supplementary material for: The ubiquitous flavonoid quercetin is an atypical KCNQ potassium channel activator
Source: Commun Biol. 2020 Jul 8;3:356. doi: 10.1038/s42003-020-1089-8 (PMC7343821; doi:10.1038/s42003-020-1089-8)
Supplement: Supplementary file 3 — Description of Additional Supplementary Files [file 42003_2020_1089_MOESM3_ESM.pdf]

Supplementary Data 1:
Source data used to generate figures 1 through 5.

Supplementary Data 2:
Source data used to generate figures 6 through 10.
